# Recent advances in the treatment of hip fractures in the elderly

**DOI:** 10.12688/f1000research.8172.1

**Published:** 2016-08-11

**Authors:** Joshua C. Rozell, Mark Hasenauer, Derek J. Donegan, Mark Neuman

**Affiliations:** 1 Department of Orthopaedic Surgery, University of Pennsylvania, Philadelphia, PA, USA; 2 Department of Anesthesiology and Critical Care, University of Pennsylvania, Philadelphia, PA, USA

**Keywords:** Hip fracture, elderly, total hip, arthroplasty, hemiarthroplasty, open reduction internal fixation

## Abstract

The treatment of hip fractures in the elderly represents a major public health priority and a source of ongoing debate among orthopaedic surgeons and anesthesiologists. Most of these injuries are treated with surgery in an expedient fashion. From the surgical perspective, there are certain special considerations in this population including osteoporosis, pre-existing arthritis, age, activity level, and overall health that contribute to the type of surgical fixation performed. Open reduction and internal fixation versus arthroplasty remain the two major categories of treatment. While the indications and treatment algorithms still remain controversial, the overall goal for these patients is early mobilization and prevention of morbidity and mortality. The use of preoperative, regional anesthesia has aided in this effort. The purpose of this review article is to examine the various treatment modalities for hip fractures in the elderly and discuss the most recent evidence in the face of a rapidly aging population.

## Introduction

The overall trend in hip fractures in the US has shown a decline over the past 10 years. Despite the rise in the aging population with a simultaneous increase in activity level, the use of bisphosphonates and decreased use of estrogens have contributed to this change, especially in women ^1^. In the global arena, hip fracture rates in Japan and China have risen because of an increase in the elderly population as well as lifestyle changes related to urbanization, and hip fractures in women occur at the highest rate in Norway, Sweden, Denmark, and Austria ^2^. However, it is estimated that by 2030 the prevalence of hip fractures will increase to 289,000 per year nationally, making these injuries a significant public health concern. Specifically, the number of hip fractures among men is projected to increase by 52%. By 2050, there will be an estimated 3.9 million hip fractures worldwide and 700,000 in the US ^3^, amounting to over $15 billion per year in medical costs ^4^. Some compounding reasons for this relative rise are that the percentage of people older than 65 years old will increase by over 80% ^5^ and that 90% of hip fractures occur in patients older than 65 years old ^6^. In a sampling of patients over the past 10 years, the distribution of hip fracture types has also changed. There has been a steep rise in the number of unstable extracapsular fractures in the elderly—that is, intertrochanteric (IT)/subtrochanteric hip fractures—while the number of intracapsular hip fractures (that is, femoral neck fractures) has remained stable ^7^. Pertrochanteric fractures in the region surrounding the greater and lesser trochanters now account for about half of all hip fractures in the elderly ^8, 9^ and although this may be due in part to osteoporosis, the underlying reasons are still not entirely clear. The high expense of treating a hip fracture is known, however. The average patient with a hip fracture spends $40,000 in the first year in direct medical costs ^1, 5^ and approximately $5,000 in each of the following years. Despite this tremendous financial burden, which includes hospital costs, rehabilitation, and nursing care, there remains a 21 to 30% risk of mortality within 1 year of sustaining a hip fracture in the elderly population ^1^, a risk up to three times higher in men compared with women ^3^.

## Epidemiology

Surgical treatment for hip fractures in the elderly represents the standard of care ^10^. Non-operative treatment has resulted in secondary fracture displacement of up to 62%; increased medical complications such as urinary tract infections, pneumonia, and deep vein thrombosis; and poor functional outcomes ^11^. Despite the high cost of surgical treatment, a recent economic analysis showed that there is actually a societal benefit to surgery compared with non-operative management; average savings per patient are $65,279 and $67,964 for displaced intracapsular and extracapsular hip fractures, respectively. This includes costs offset by continued nursing home care and long-term medical costs ^6^. Non-operative management is typically reserved for critically ill patients not medically stable for surgery or non-ambulatory patients. Although the goals of treatment in young patients are anatomic reduction and stable fixation ^11^, the purpose of fixation in the elderly population focuses more on the restoration of function and a decrease in secondary complications. Treatment can be accomplished by various methods but most commonly includes open reduction and internal fixation (intramedullary versus extramedullary) or arthroplasty ^12, 13^.

## Surgical timing

Regardless of the treatment method, there has been a push toward surgery within 48 hours of hospital admission. Several studies have examined the correlation between surgical timing and subsequent morbidity and mortality in elderly patients ^14– 16^. In one meta-analysis of over 250,000 patients, an operative delay beyond 48 hours resulted in an absolute risk of 41% increased 30-day mortality rate and a 32% increased odds of 1-year mortality ^15^. Similarly, in a prospective observational study of 5683 male veterans over the age of 65 with hip fractures, a surgical delay of greater than 4 days resulted in a higher mortality risk, especially in patients in a higher pre-operative risk stratification group ^16^. Therefore, hip fracture surgery in an elderly patient should be performed expediently with focused consideration of comorbidities and post-operative reduction in pain and return to functioning in order to maximize outcomes.

## Intertrochanteric hip fractures

**Figure 1.**
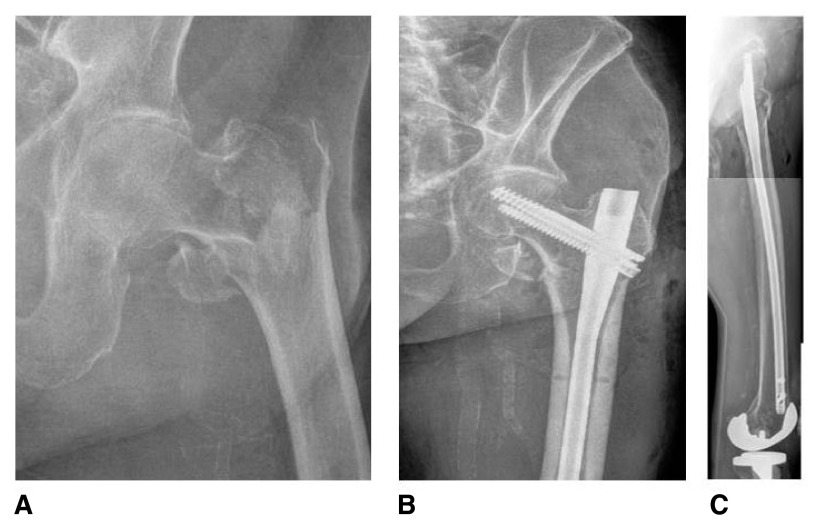
Pre-operative anteroposterior ( **A**) and post-operative anteroposterior ( **B**) and lateral ( **C**) radiographs of a 94-year-old male who underwent long intramedullary fixation for a left unstable intertrochanteric hip fracture.

**Figure 2.**
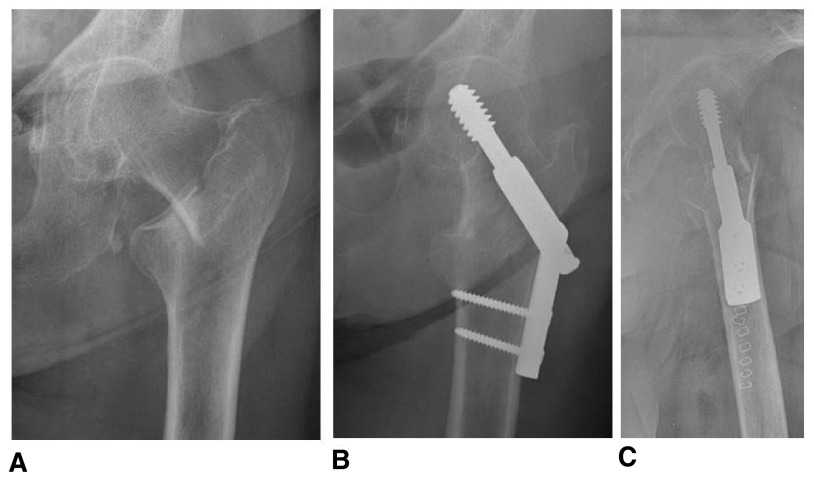
Pre-operative anteroposterior ( **A**) and post-operative anteroposterior ( **B**) and lateral ( **C**) radiographs of an 87-year-old female who underwent sliding hip screw fixation for a left stable intertrochanteric hip fracture.

Since its introduction in the 1980s, cephalomedullary fixation for IT fractures in the elderly has gained popularity. Aside from the theoretical advantage of being less invasive and biomechanically superior ^17– 19^, these devices have been advocated in cases of unstable fracture patterns such as reverse obliquity, lateral wall incompetence, subtrochanteric extension, and medial calcar disruption ^8, 20, 21^. A review of reverse obliquity fractures in a large Scandinavian patient registry has corroborated the use of the nail, demonstrating a higher re-operation rate (6.4% versus 3.8%) at 1 year in the sliding hip screw (SHS) group compared with the intramedullary nail group, as well as a higher pain score and lower satisfaction rating. The lower overall numbers of re-operation may be due to the addition of a trochanteric stabilization plate to resist femoral shaft medialization, but this contributes to increased operative time and may add technical complexity ^22^. In a cross-sectional survey distributed to practicing orthopedic surgeons, Niu *et al*. found that 68% of the 3786 respondents across all levels of experience primarily used cephalomedullary devices for IT fractures for reasons such as ease of use, potential improvements in functional outcome, and biomechanical advantage ^8^. Using a newer-generation long or short nail does not seem to have an effect on re-operation rate, risk of periprosthetic fracture, or mortality rate ^18, 20^. One potential advantage of using a long nail in the elderly population is that it disperses intramedullary forces and limits diaphyseal stress risers in already-weak bone. If the device is used as an internal splint of the entire long bone endoskeleton, the risk of periprosthetic fracture may be mitigated ( Figure 1). However, a cephalomedullary nail does cost approximately $900 to $1500 more than an SHS. In a cost analysis of the two implants, Swart *et al*. found that for stable IT fractures, the SHS was more cost effective ^23^. This cost may be partially offset by increased length of stay after SHS fixation ^24^, but for stable or minimally displaced fractures, the SHS remains a successful treatment ( Figure 2) ^25^.

## Subtrochanteric hip fractures

The management of subtrochanteric fractures is challenging because of the inherent instability of the fracture pattern and the large muscular deforming forces on the proximal and distal fragments. Flexion and external rotation of the proximal fragment, combined with potential comminution of the calcar and adductor forces medializing the femoral shaft, render reduction difficult. Both intramedullary and extramedullary devices have been used for these injuries. Among three level I studies of elderly patients included in a meta-analysis, the data of Kuzyk *et al*. favor the more reliable use of intramedullary devices ^26^. In a more recent meta-analysis comparing intramedullary and extramedullary fixation for subtrochanteric femur fractures in the elderly, there was an 83% lower relative risk of revision in the intramedullary nail group and 76% lower rate of fixation failure in the intramedullary group ^27^. With regard to other factors, there was no difference in intra-operative blood loss, case length, and post-operative complications. Moreover, in patients with comorbidities or osteoporotic bone, intramedullary fixation for subtrochanteric femur fractures is preferred.

## Arthroplasty for hip fractures

Much of the recent advancement in the treatment of hip fracture and specifically femoral neck fracture surgery in the elderly has taken place in the realm of arthroplasty. Several questions arise when considering joint replacement for fracture: is a total hip arthroplasty (THA) or hemiarthroplasty (HA) a better option? If HA is performed, should a unipolar or bipolar device be used? Should the femoral implant be cemented? Again, the overarching goal is to allow the fastest recovery with the lowest complication risk.

Bone quality plays a large role in the success of hip fracture fixation. Patients with osteoporosis have a 30% increased risk of nonunion when internal fixation is the treatment ^11^. This has resulted in a plethora of research focused on arthroplasty as an alternative to replace the osteoporotic bone. A short-term meta-analysis by Gao *et al*. evaluated 4508 patients with femoral neck fractures over the course of a 5-year period ^13^. They reported that compared with internal fixation, arthroplasty reduced the risk of major complications and the incidence of re-operation. Pain relief and function were also improved, but mortality rates were similar. This emphasizes the tremendous setbacks in quality of life and longevity sustained after a hip fracture regardless of treatment. In a longer-term meta-analysis with at least 4 years of follow-up, Jiang *et al*. similarly found no difference in mortality between the internal fixation and arthroplasty groups but did find improved re-operation risk and mid-term functional improvements after arthroplasty ^28^. Despite the larger incision and dissection required for arthroplasty, there was no increased incidence of wound infection. Unique to each treatment modality, dislocation was higher in the arthroplasty group, whereas aseptic necrosis and nonunion were more prevalent in the internal fixation group. Finally, the longest-term meta-analysis of a 15-year minimum by Johansson reported a 55% failure rate with internal fixation compared with a 5% failure rate after arthroplasty ^29^. In this group of 146 hips in patients who were at least 75 years old, there was also no difference in mortality between the groups. Other studies have also found a higher risk of revision surgery after internal fixation and recommend arthroplasty for healthy, lucid elderly patients ^30– 33^. Although the rate of re-operation is lower, it should be recognized that arthroplasty introduces a distinct subset of complications, namely dislocation, aseptic loosening, infection, and wound complications.

## Total hip arthroplasty vs hemiarthroplasty

**Figure 3.**
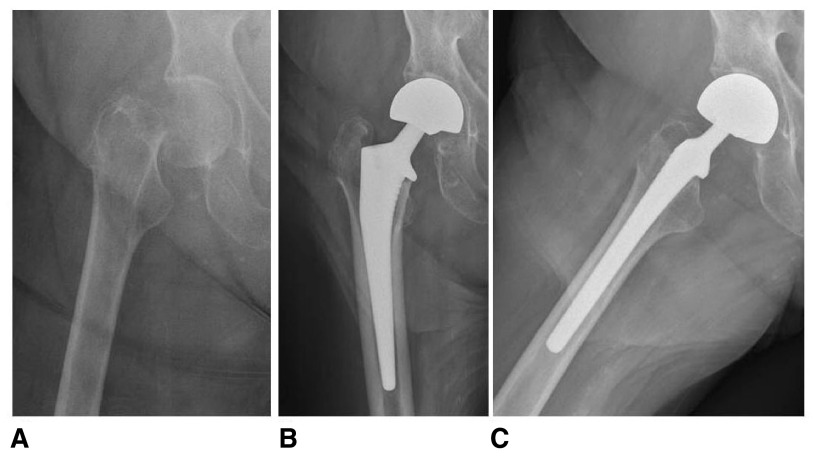
Pre-operative anteroposterior ( **A**) and post-operative anteroposterior ( **B**) and lateral ( **C**) radiographs of a 91-year-old female who underwent an uncemented HA for a displaced, right subcapital femoral neck fracture.

**Figure 4.**
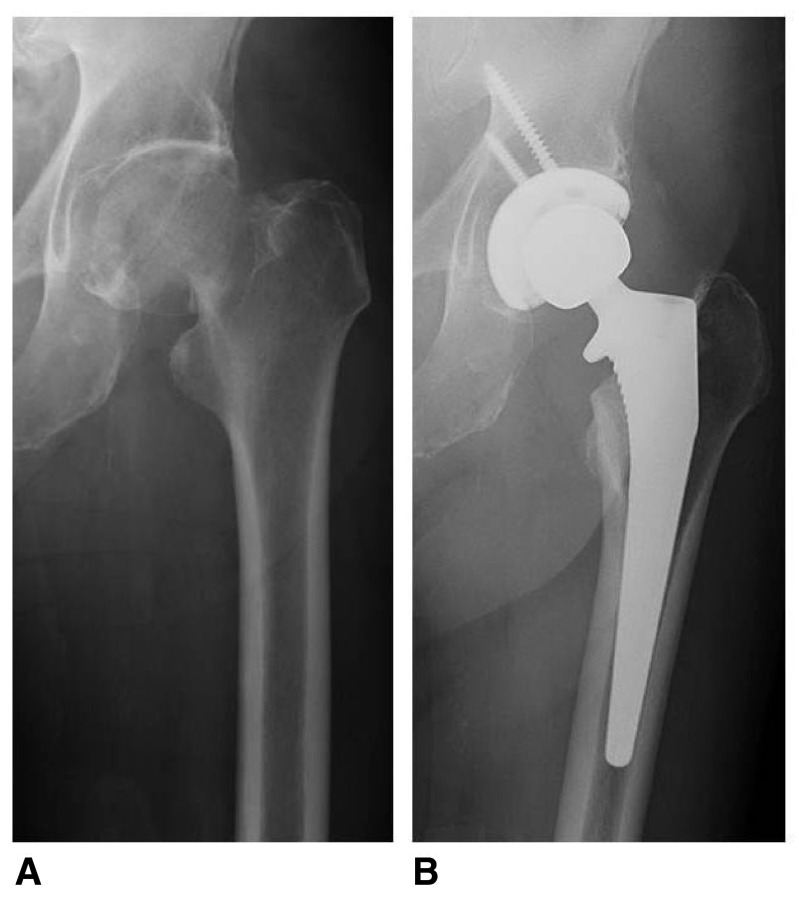
Pre-operative ( **A**) and post-operative ( **B**) anteroposterior radiographs of an 81-year-old male who underwent a total hip arthroplasty for a displaced, left subcapital femoral neck fracture.

Once arthroplasty is the decided treatment, the surgeon must decide whether a total hip replacement or a partial replacement is warranted. The Hip Fracture Evaluation with Alternatives of Total Hip Arthroplasty versus Hemiarthroplasty (HEALTH) trial is underway to attempt to clarify this debate. This is a prospective, multicenter, randomized trial comparing THA versus HA in patients at least 50 years old who sustain displaced femoral neck fractures. The primary outcome is revision surgery rates at 2 years post-operatively, and functional scores, quality of life, and complications are secondary outcome measures ^34^. Although this represents the largest study of its kind, others have elucidated the differences in treatment options. Pre-operative considerations include the presence of pre-existing osteoarthritis, medical comorbidities, mental status, and functional demand. Lower-demand patients may be more suitable for a HA ( Figure 3) given the lower risk of dislocation, elimination of problems associated with acetabular reaming and version, and decreased operative time and blood loss; patients with cognitive dysfunction may not be able to comply with certain hip precautions, leading to a higher rate of dislocation. Higher-demand patients may avoid re-operation secondary to acetabular erosion from a large HA head if they undergo a THA ( Figure 4). The American Academy of Orthopaedic Surgeons Clinical Practice Guideline (AAOS CPG) on hip fractures in the elderly cites moderate evidence to support a benefit to THA in properly selected patients with displaced femoral neck fractures. However, the benefits of lower pain scores and lower revision rates for acetabular wear may be confounded by a selection bias in that more active, healthy individuals undergo a THA compared with HA ^35^. Zi-Sheng *et al*. conducted a meta-analysis showing that compared with HA, THA has a lower long-term risk of re-operation rates at 13 years post-operatively but not mortality ^4^. However, the rate of dislocation was higher in the THA group (17.2% versus 4.5%). Similar findings were reported by Hopley *et al*. in their systematic review of 1890 patients more than 60 years old; the authors cited a 4.4% risk of re-operation difference in THA compared with HA as well as better Harris Hip Score (HHS) ratings up to 4 years post-operatively ^36^. Several other meta-analyses concur with these findings, although there are inherent limitations in using the HHS for evaluation ^37– 39^.

## Unipolar vs bipolar hemiarthroplasty

The second point in the HA decision tree is a unipolar or bipolar articulation. Bipolar femoral heads were initially introduced to decrease acetabular wear and increase hip range of motion to decrease dislocation rate ^40– 42^. The added articulation may also make conversion to a total hip replacement easier. In a randomized controlled trial with 4-year follow-up, Inngul *et al*. evaluated 120 patients over the age of 80 randomly assigned to unipolar versus bipolar cemented HA ^43^. Patients in the unipolar group displayed a higher incidence of acetabular erosion at 1 year ^44^, but no significant differences were seen at the 2- and 4-year time points ^43^. There was also no difference seen in functional scores or re-operation rates. In another randomized trial, Kanto *et al*. showed that patients in the unipolar group (n = 88) had a higher dislocation rate than the bipolar group (n = 87), but this difference did not translate into increased revision rates by 8 years post-operatively ^45^. There were no significant differences in ambulatory function, early acetabular erosion, or mortality. The long-term outcome similarities are thought to be due to a unitization of the bipolar articulation over time, thus rendering the construct functionally unipolar ^40^. Despite the risk of acetabular erosion, the risk of conversion of a bipolar HA to a THA is low and is similar to the rate of conversion from a unipolar construct. One study reported that of the 164 included patients, only four underwent a conversion after 1 year, and only one (0.6%) was performed for groin pain ^46^. Von Roth *et al*. analyzed 376 cemented bipolar HAs and found that the rate of conversion to THA at 20 years was only 3.5% and that only 1.4% were due to cartilage wear ^47^. Even though an increase in patient age is commensurate with decreased activity level and thus decreased cumulative wear over time, no living patient at the endpoint of follow-up showed cartilage wear or implant loosening ^47^. In the most recent meta-analysis, Jia *et al*. again found a lower acetabular erosion rate within the first year for bipolar articulations but no other significant differences with regard to mortality, complications, and functional outcome scores, challenging the hypothesis that the bipolar prosthesis produced a less painful arthroplasty and improvement in quality of life ^42^. This review does not explore the rates of conversion to THA.

## Femoral stem fixation: cemented vs uncemented

**Figure 5.**
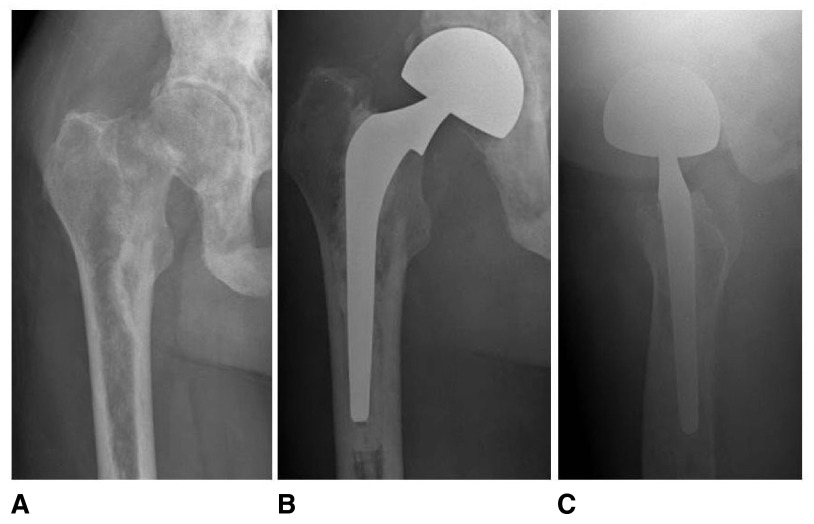
Pre-operative anteroposterior ( **A**) and post-operative anteroposterior ( **B**) and lateral ( **C**) radiographs of a 79-year-old male with metastatic prostate cancer who underwent a right cemented hip hemiarthroplasty for a pathologic femoral neck fracture.

In both HA and THA, femoral stem fixation remains an area of controversy. The AAOS CPG on hip fractures in the elderly put forth moderate evidence in support of cemented femoral stems in patients undergoing arthroplasty for femoral neck fractures ^35^. Using cemented fixation results in an overall decrease in periprosthetic fracture risk ^30, 48^ and an improved rate of loosening. In addition, cement interdigitates well in osteoporotic bone ^49^ and antibiotics may be mixed into the cement for infection prophylaxis ( Figure 5). Potential disadvantages of cement include increased operative time, difficulty with extraction in the case of revision, and the risk of fat embolus or bone cement implantation syndrome (BCIS). With a steep learning curve, improper cementation technique may also result in a varus femoral stem, thus putting the prosthesis at risk for failure.

Data from 19,669 patients in a Scottish registry were evaluated to investigate the mortality associated with cemented fixation. Cemented HA was associated with a small but significantly higher rate of mortality than uncemented HA on day zero and up to day one ^50^. Age was also an independent predictor of mortality. In specific patient populations, cement is not advised. The monomer from the cement enters the bloodstream during pressurization and may cause hypotension, thus decreasing cardiac output and overloading heart function in patients with heart disease. Patients with decreased pulmonary reserve are at risk for BCIS. If cementation is undertaken in these patients, it is important to make the anesthesia team aware of when the cement is being pressurized ^51^. Lastly, elderly patients have a higher fat-to-marrow ratio, thus increasing the risk of fat embolism.

When cementation is successful, there may be a lower re-operation rate in the long term. At 1-year follow-up, Deangelis *et al*. found no difference in functional outcomes or acute complications ^52^ when comparing uncemented and cemented cohorts. However, comparing re-operation rates among elderly patients undergoing cemented versus uncemented HA, Viberg *et al*. found that the cemented cohort had a decreased hazard ratio and a superior long-term implant survival rate after 3 years compared with the uncemented group ^53^. A Norwegian registry of 11,116 HAs evaluated in a prospective observational study showed that at 5-year follow-up, uncemented HA had a 2.1-fold increased risk of revision, most commonly for periprosthetic fracture. In keeping with the intra-operative complications of cementation noted above, there was a higher risk of intra-operative mortality in their cemented group, although longer-term mortality risk was not significantly different ^30^. These studies, however, do not account for the fact that patients in the cemented group may have inferior bone quality, which may be a surrogate marker for other comorbidities. In a study by Taylor *et al*., HA with a cemented implant provided a comparable outcome to the uncemented group in patients without severe cardiac disease, although there was a trend toward better function and mobility in the cemented group ^49^. Both groups, however, displayed an approximately 18 to 24% decrease in independence when compared with their pre-operative level of functioning ^49^. Thus, it is important to evaluate the patient in terms of functional capacity but also medical stability prior to deciding whether a cemented prosthesis is the best option. In otherwise-healthy, elderly patients with osteoporosis, cemented HA is a good option in terms of post-operative pain and re-operation rates.

## Anesthesia considerations in hip surgery

A comprehensive pre-operative evaluation by the anesthesia team also plays a large role in the type of anesthesia used during surgery and has significant implications in the patient’s immediate post-operative recovery. The use of spinal versus general anesthesia, the two most common anesthetic modalities in hip fracture surgery, and the use of peripheral nerve blocks have entered into the research spotlight over the past several years. Prompting a large proliferation of research in this area, a 2011 review by the UK National Clinical Guideline Center examined a group of 22 trials and concluded that no recent randomized trial had been able to fully address or appreciate the differences between general and neuraxial anesthesia for hip fractures ^54^. Few studies since that time have shown a trend toward lower delirium rates, complications, and mortality associated with regional anesthesia ^55, 56^, and other older studies were unable to demonstrate any significant differences in mortality beyond 2 months ^57– 61^. Furthermore, these studies have influenced the opinions of various subspecialty associations that continue to spur debate in this arena ^35^. An important consideration arises in patients with more significant illness or comorbidities who may be subject to a selection bias favoring regional anesthesia, potentially overestimating the association between specific anesthesia techniques and patient outcomes ^62^. To this end, the REGAIN trial (Regional versus General Anesthesia for Promoting Independence after Hip Fracture, NCT02507505) now underway is examining the effects of general versus spinal anesthesia on post-operative mobility and overall health status following hip fracture surgery in elderly patients. REGAIN will randomly assign 1600 patients across about 30 hospitals in the US and Canada to spinal versus general anesthesia over 3.5 years, and the goal is to provide a stronger evidence base going forward to inform treatment decisions among patients with hip fracture.

The addition of peripheral nerve blocks has aided in pain reduction after hip fractures as well as limiting post-operative psychological complications such as delirium in the elderly population. Seen in approximately 10 to 16% of elderly surgical patients, delirium is associated with delayed rehabilitation, prolonged hospital stay, and poorer functional outcomes ^63^. A 2002 Cochrane review of eight trials demonstrated that nerve blocks resulted in a reduction in the amount of oral and parenteral analgesics administered post-operatively ^64^. In corroboration, several studies have reported a reduction in pre-operative pain or delirium (or both) following either femoral nerve or fascia iliaca blockade ^63, 65^. Foss *et al*. reported on 48 patients with hip fractures randomly assigned to mepivacaine fascia iliaca blocks or placebo injections ^65^. Those in the placebo group received intramuscular rescue doses of morphine. The authors reported that pain relief was superior at all time points in patients who received the block and that sedative risk was increased in the placebo group receiving morphine ^65^. Patients may even be able to receive these blocks in the emergency department almost immediately after they arrive, and this may decrease their total narcotic use prior to surgery. In a small, prospective observational study, elderly patients receiving blocks in the emergency department reported a decrease in pain, up to 67%, only 30 minutes after the block was administered. The pain scores were unchanged up to 4 hours post-operatively ^66^. Regarding the efficacy of two blocking options, Newman *et al*. conducted a comparison of pre-operative femoral nerve versus fascia iliac compartment blocks in patients with femoral neck fractures ^67^. The authors showed that though requiring more time to administer and slightly more expensive, femoral nerve blocks result in a greater reduction in pain according to the visual analogue scale.

## Post-operative care

Post-operatively, hemodynamic stability serves as an additional barometer of earlier participation in rehabilitation and potentially a quicker hospital discharge. Perhaps the most robust study in this area is a follow-up to the trial of transfusion requirements in critical care patients. A 2011 randomized controlled trial of 2016 patients across 47 clinical sites compared a conservative transfusion threshold (8 g/dL) versus a more liberal one (10 g/dL) in elderly patients with a history of cardiovascular disease (or risk factors) and a hip fracture. Patients in the liberal group were given red blood cell transfusions to maintain a hemoglobin level of 10 g/dL. Patients in the conservative group required a hemoglobin level of 8 g/dL or below and symptoms of anemia to receive a transfusion. The primary outcome measure was the ability to walk 10 feet unassisted at 60 days’ follow-up. Carson *et al*. showed that there was no evidence to support a more liberal transfusion strategy in patients with hip fracture ^68^. This study not only has cost implications but may allow patients to be mobilized more quickly, decreasing hospital length of stay and complications associated with prolonged hospitalization.

As the proportion of elderly patients continues to rise, most orthopedic surgeons will treat a patient with a hip fracture in their career. In the face of a changing healthcare landscape and bundled payments, optimization of surgical treatment to promote early mobilization and decreased hospital stay are paramount. Of paramount importance is the integration of a multidisciplinary approach to hip fractures. The introduction of the orthogeriatrician has been helpful in facilitating early surgery, immediate mobilization, prevention and management of delirium, pain, and malnutrition, and an integrated and multidisciplinary approach to post-operative care ^69^. Recent advances in the literature have elucidated indications for fixation and arthroplasty. Although there is still controversy in the nuances of each procedure, there are several overarching themes. Pre-operative regional blocks and post-operative multimodal anesthesia have been shown to be highly effective in pain management and narcotic reduction. Further studies should focus on elucidating these trends post-operatively. Displaced femoral neck fractures in active, otherwise-healthy elderly patients are best treated with a THA, especially if they have pre-existing groin pain consistent with osteoarthritis. Lower-demand patients may be better suited to a HA, and a cemented, unipolar arthroplasty is the current treatment of choice. With further advances in implant design, risks such as component wear and dislocation may be mitigated. For unstable IT or subtrochanteric fractures, the fracture pattern largely determines the implant choice.
